# Strategies for Enzymatic Inactivation of the Veterinary Antibiotic Florfenicol

**DOI:** 10.3390/antibiotics11040443

**Published:** 2022-03-25

**Authors:** Marik M. Müller, Ruslan Nedielkov, Katja M. Arndt

**Affiliations:** 1 Molecular Biotechnology, Institute of Biochemistry and Biology, University of Potsdam, Karl-Liebknecht-Str. 24-25, 14476 Potsdam, Germany; marik.m.mueller@gmail.com; 2 Analytical Chemistry, Institute of Chemistry, University of Potsdam, Karl-Liebknecht-Str. 24-25, 14476 Potsdam, Germany; ruslan.nedielkov@uni-potsdam.de

**Keywords:** aquaculture, antibiotic inactivation, enzyme optimization, enzymatic inactivation, florfenicol, immobilization, industrial farming

## Abstract

Large quantities of the antibiotic florfenicol are used in animal farming and aquaculture, contaminating the ecosystem with antibiotic residues and promoting antimicrobial resistance, ultimately leading to untreatable multidrug-resistant pathogens. Florfenicol-resistant bacteria often activate export mechanisms that result in resistance to various structurally unrelated antibiotics. We devised novel strategies for the enzymatic inactivation of florfenicol in different media, such as saltwater or milk. Using a combinatorial approach and selection, we optimized a hydrolase (EstDL136) for florfenicol cleavage. Reaction kinetics were followed by time-resolved NMR spectroscopy. Importantly, the hydrolase remained active in different media, such as saltwater or cow milk. Various environmentally-friendly application strategies for florfenicol inactivation were developed using the optimized hydrolase. As a potential filter device for cost-effective treatment of waste milk or aquacultural wastewater, the hydrolase was immobilized on Ni-NTA agarose or silica as carrier materials. In two further application examples, the hydrolase was used as cell extract or encapsulated with a semi-permeable membrane. This facilitated, for example, florfenicol inactivation in whole milk, which can help to treat waste milk from medicated cows, to be fed to calves without the risk of inducing antibiotic resistance. Enzymatic inactivation of antibiotics, in general, enables therapeutic intervention without promoting antibiotic resistance.

## 1. Introduction

The therapeutic use of antibiotics is an important milestone in medicine that has drastically reduced the mortality rate of many bacterial infections. However, the increased use of antibiotics in humans as well as in animal husbandry has resulted in an alarming rise of antimicrobial resistance (AMR). As a result, nowadays many multi-drug resistant bacteria exist threatening human health. In 2015 in Germany alone, almost 55,000 people contracted an infection caused by antibiotic-resistant bacteria, 2400 of whom died. In the EU, more than 33,000 people die every year from infections caused by antibiotic-resistant bacteria [1,2]. A recent meta-analysis estimated for 2019 worldwide 1.27 million deaths attributable to bacterial AMR [3].

The antibiotic florfenicol (FF) is a fluorinated synthetic analog of thiamphenicol and chloramphenicol with the same mechanism of action, blocking protein synthesis by inhibiting the peptidyl transferase activity of bacterial ribosomes [4]. As a broad-spectrum antibiotic, florfenicol is used worldwide in large quantities as a veterinary drug and feed additive for aquaculture, poultry, and livestock [5,6]. Chile, the world’s second largest salmon producer, used from 2007 to 2012 over 5500 tons of antibiotics in salmon farming. In 2017 alone, almost 400 tons of antibiotics were used, with florfenicol accounting for over 90% [7,8]. One particular problem is that florfenicol is poorly absorbed by the organism, resulting in 40–90% being excreted, entering the environment and promoting the development of AMR [9,10,11,12]. In Vietnam, florfenicol is the major antibiotic for swine and chicken, which are treated with about 200 tons of florfenicol per year [6]. The resulting waste releases large quantities of this antibiotic into the environment, which significantly contributes to the rise of AMR [13]. Over 80% of the enterobacteria *Escherichia coli* isolated from California diaries showed resistance to florfenicol [14], and in China, more than 60% of a Salmonella enterica strain, a pathogen of cattle, poultry and potentially humans, was florfenicol resistant [15]. Additionally, in Germany, 6.2 tons of florfenicol were supplied to veterinarians in 2019 [16]. An additional problem is the treatment of cows with antibiotics during lactation, resulting in the production of waste milk containing antibiotic residues. Florfenicol is detectable in the milk for up to 30 days after treatment and is not inactivated by heat treatment, such as pasteurization or processing, such as cheese making [17]. Waste milk is not allowed to be sold for human consumption, but it is often fed to calves, leading to increased numbers of antibiotic-resistant bacteria in their intestinal and respiratory tracts [18,19,20,21]. 

Several florfenicol resistance mechanisms have been described including different efflux pumps, an rRNA methylase that prevents binding of florfenicol to the ribosome, as well as ribosome-protecting proteins [5,22,23]. Importantly, the majority of these resistance genes are not selective to florfenicol leading to the development of multidrug-resistant bacteria [24,25,26,27]. To date, only one enzyme (hydrolase, EstDL136) has been described to be capable of cleaving chloramphenicol and florfenicol, albeit with low efficiency [28,29].

Despite being used only in veterinary medicine, there is an alarming increase of florfenicol resistance in clinical isolates around the world, often leading to multi-drug resistance. A recent study reported a dramatic increase of florfenicol-resistant invasive non-typhoidal Salmonella isolated from clinical patients [30]. The rate increased from 0% in 2007–2009 to over 30% in 2015 and 2016. Even more alarming, 94% of the strains the authors investigated were multi-drug resistant and the other 6% resistant to two antimicrobial agents. Another study analyzed 430 clinical *Pseudomonas aeruginosa* isolates and found 94.65% to be resistant to florfenicol [31]. Such increasing resistances threaten food production and human health, and the world health organization ranks antimicrobial resistance as “one of the most urgent health threats of our time” [32]. 

While antibiotic use should be limited, it is very important to also develop strategies for antibiotic inactivation in waste products to decrease the risk of AMR development. To devise an efficient and environmentally-friendly way to inactivate florfenicol, we optimized the hydrolase EstDL136 for cleavage of the antibiotic florfenicol. For repeated use, the hydrolase was immobilized on carrier materials. Florfenicol inactivation was demonstrated in saltwater and cow milk establishing potential use scenarios, such as florfenicol inactivation in waste milk from medicated cows, which enables feeding this milk to calves without the risk of inducing antibiotic resistance. Such an enzymatic inactivation of antibiotics, in general, enables therapeutic intervention without promoting antibiotic resistance.

## 2. Results

### 2.1. Ability of the Hydrolase Estdl136 to Confer Resistance to Chloramphenicol and Florfenicol

**Figure 1:**
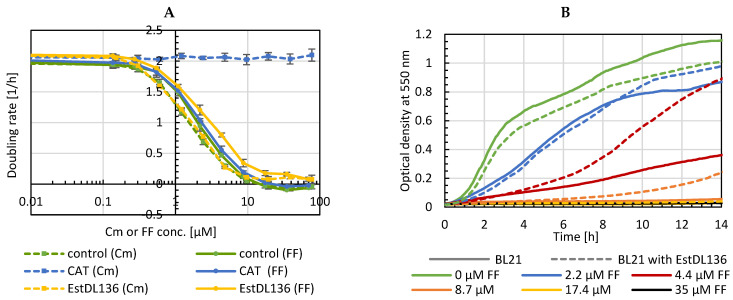
Growth assays in Cm or FF of *E. coli* expressing CAT or EstDL136. (**A**) Doubling rates were calculated from early logarithmic growth phase (control = BL21 without plasmid; *n* = 4). (**B**) Growth curves of BL21 without (straight line) or with hydrolase (dashed line) in media with different FF concentrations.

The effect of florfenicol (FF) on susceptible *E. coli* was compared to chloramphenicol (Cm) by analyzing bacterial growth at different antibiotic concentrations. A similar effect for both antibiotics was observed (Figure 1A). Since it was published that the hydrolase EstDL136 can also cleave florfenicol [29], the gene was cloned on an expression plasmid, and the doubling rate of cells expressing either hydrolase or the resistance marker chloramphenicol acetyltransferase (CAT) was compared in the presence of different antibiotic concentrations. Figure 1A shows that bacteria carrying the resistance marker CAT grow in Cm containing media but are sensitive to FF. This can be explained by CAT inactivating Cm but not FF as the fluoride atom prevents acetylation. Bacteria expressing the hydrolase enzyme are sensitive to Cm and show only marginal tolerance to FF. While initial doubling rates are similar, hydrolase expressing bacteria are able to grow at low FF concentrations after extended incubation time (Figure 1B), indicative of inactivation of FF with low efficiency.

### 2.2. Optimization of the Hydrolase Estdl136 for Increased Florfenicol Inactivation

**Figure 2:**
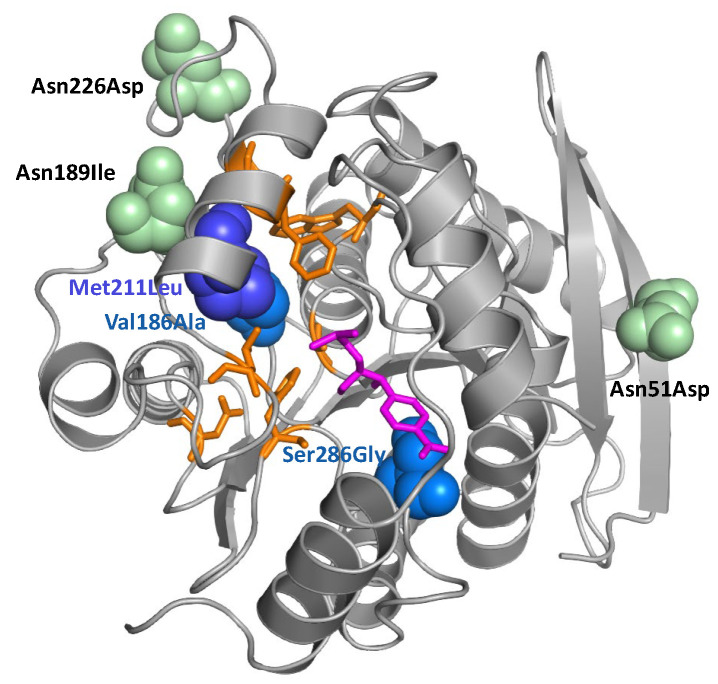
Cartoon backbone representation of the hydrolase structure. Pink: chloramphenicol; orange: amino acids involved in Cm binding and cleavage according to [33]; blue: mutations near the active center; green: further mutations. The figure was generated using Pymol [34].

**Figure 3:**
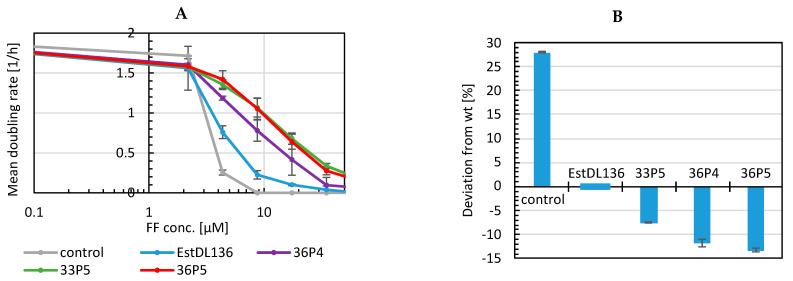
Growth assay of *E. coli* expressing wild-type hydrolase EstDL136 and mutants after retransformation (**A**) Comparison of selected mutants in liquid media containing different FF concentration (*n* = 5 (EstDL136); *n* = 4 (33P5, 36P4); *n* = 6 (36P5); *n* = 2 (control = BL21 without plasmid)); (**B**) Inhibition zone assay. The size of the inhibition zone was measured from scanned plates and calculated as a percentage deviation from cells expressing the wild-type hydrolase EstDL136.

To improve the activity of the hydrolase, a combinatorial approach combined with selection under increasing FF pressure was chosen. Mutations were introduced by error-prone PCR, and metabolic selection was performed at increasing FF concentrations in liquid media and on agar plates. Enriched clones were sequenced (Table 1), identified mutations located in the hydrolase’s three-dimensional structure (Figure 2) and tested in bacterial growth assays (Figure 3). 

Mutations were distributed across the hydrolase sequence and structure (Table 1 and Figure 2). On average, three to five mutations per clone were found (including silent mutations), some of them in several clones. In addition to the amino acid mutations listed in Table 1, mutant 33P5 also carried two silent mutations at amino acid positions Ile88 (ATC to ATT) and Gly216 (GGC to GGG), while mutant 36P5 carried one silent mutation at Ala244 (GCT to GC C). Of special interest is the mutation Valine to Alanine at position 186 (Val186Ala) as it was found in all the clones analyzed. The mutation from methionine to leucine at position 211 (Met211Leu) also occurred frequently. Interestingly, both mutations are close to the Chloramphenicol binding site in the three-dimensional protein structure (Figure 2).

To compare individual mutants, growth assays were performed (Figure 3 and Figure S1) in media supplemented with increasing florfenicol concentrations. As the lag phases until the onset of the exponential growth phases varied considerably at different florfenicol concentrations, a mean doubling rate, referring to the time in which the cells have doubled twice, was calculated. It was previously observed that *E. coli* strain RV308 is able to adapt to low florfenicol concentrations (Figure S2). To rule out a similar adaptation of *E. coli* BL21 during the selection process, plasmids from selected clones were isolated and retransformed into fresh BL21 cells before repeating the growth assays.

Figure 3A shows that *E. coli* harboring the selected mutants grew significantly better than *E. coli* expressing the wild-type hydrolase even after retransformation. Importantly, there was no difference to the growth assay before retransformation. In addition, bacterial growth was tested on agar plates using an inhibition zone assay. For this purpose, BL21 cells harboring different hydrolase mutants were plated on agar plates, and a filter disk soaked with florfenicol was placed in the center of each agar plate (Figure 3B). The size of the inhibition zone was measured from scanned plates and calculated as a percentage deviation from the wild-type hydrolase. To minimize measurement errors, three independent measurements were taken, and the mean and standard deviation were calculated.

### 2.3. Characterization of the Hydrolase Mutant 36P5

**Figure 4:**
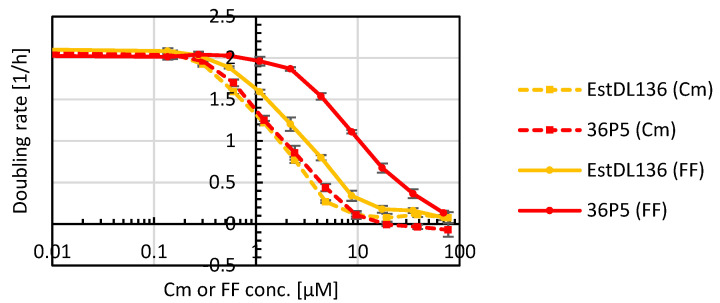
Comparison of wild-type hydrolase EstDL136 and mutant 36P5. Growth assay in Cm or FF of *E. coli* expressing either EstDL136 or mutant 36P5 (*n* = 4).

The best performing mutant 36P5 was analyzed further. To assess if the improvement of the hydrolase was specific for FF, *E. coli* expressing wild-type hydrolase EstDL136 or mutant 36P5 were grown in media with different concentrations of Cm or FF (Figure 4). Importantly, the optimized mutant 36P5 conferred better tolerance to FF but not to Cm, indicating that the obtained mutations were indeed specific for improved FF cleavage.

**Figure 5:**
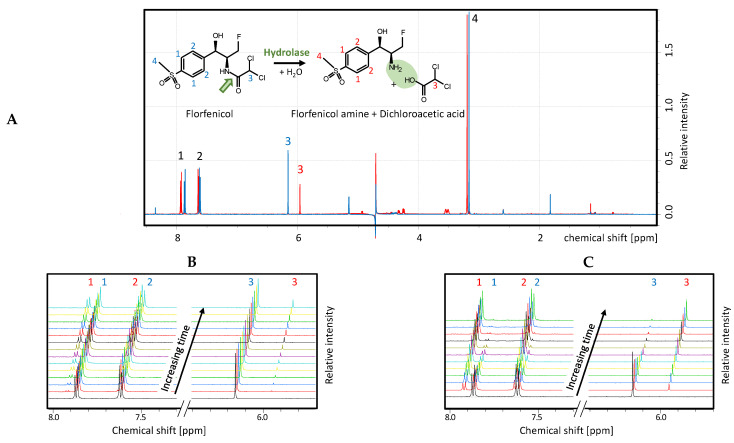
Florfenicol cleavage visualized by NMR spectroscopy. (**A**) Chemical reaction scheme of florfenicol cleavage and corresponding ^1^H-NMR spectrum before (blue) and after (red) addition of hydrolase. The assignment of the NMR signals to the corresponding protons in the chemical structural formula is indicated by numbers; (**B**,**C**) excerpts from the NMR spectra recorded every 4 min (colored lines) after addition of (**B**) wild-type hydrolase EstDL136 and (**C**) mutant 36P5 to florfenicol.

Since bacterial growth assays provide only an indirect measure of florfenicol cleavage, nuclear magnetic resonance (NMR) spectroscopy was used to directly visualize florfenicol cleavage (Figure 5A). Wild-type hydrolase and the best selected mutant 36P5 were expressed and purified by immobilized metal-ion affinity chromatography (IMAC) and size exclusion chromatography (SEC). Each enzyme was added to a florfenicol solution at a defined enzyme to substrate ratio. ^1^H-NMR spectra focusing on the educt and product were recorded before and after treatment with wild-type or mutant hydrolase (Figure 5A). 

To compare the florfenicol cleavage mediated by the wild-type or mutant hydrolase, NMR measurements were taken every four minutes over a period of one hour. Figure 5B,C show a section of these measurements. Each color represents a spectrum, starting four minutes after enzyme addition (lowest black curve), and then every subsequent four minutes. For a direct comparison of the catalytic activity, the wild-type hydrolase EstDL136 (Figure 5B) and the mutant 36P5 (Figure 5C) were used at the same concentration, and the cleavage of florfenicol was measured at equal time intervals. 

A comparison of the peak intensities revealed that after one hour the peaks from the educt florfenicol were no longer visible for the mutant (Figure 5C) but did not decrease much for the wild-type hydrolase (Figure 5B). Conversely, the newly formed peaks of the products were significantly larger for the mutant than for the wild-type hydrolase, indicating that the wild-type was not able to completely convert the florfenicol in the observed time span. The reaction of the wild-type hydrolase was observed further, and only after more than three hours the peaks of the educts almost completely disappeared (data not shown). Approximately eight times the amount of wild-type hydrolase had to be used in order to obtain a similar cleavage rate as for the mutant. These experiments demonstrate that the enzyme activity of the mutant 36P5 was significantly improved compared to the wild-type hydrolase.

### 2.4. Hydrolyase Immobilization

For effective and economic inactivation of florfenicol in various waste products, the hydrolase should be reusable several times. This is most easily achieved by immobilization on a carrier material. Immobilization allows the hydrolase to be incubated with florfenicol-contaminated solutions and then easily separated for further use. Simple immobilization without prior purification of the hydrolase is possible by using specific tag sequences, such as His_6_-tag or silica-tag fused to the enzyme at the genetic level. For this purpose, the improved hydrolase mutant 36P5 was tested as a fusion protein with three different tags in different fusion arrangements. For visualization of the immobilization procedure and stability, a further fusion with the green fluorescent protein GFP fusion was used.

**Figure 6:**
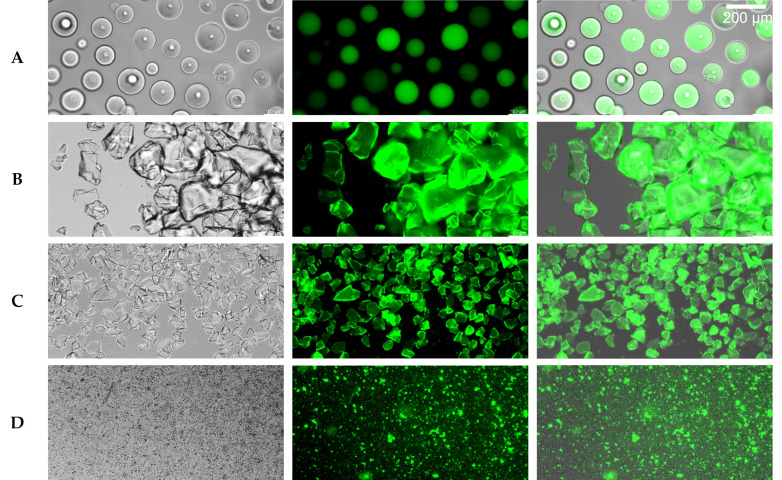
Microscope images of the immobilized hydrolase mutant 36P5. (**A**) Ni-NTA material; (**B**) silicic acid; (**C**) silica gel; (**D**) grinded sand. The figure shows the bright field (left), fluorescence (center) and superimposed images (right).

Immobilization of the hydrolase on Ni-NTA material was achieved by expressing the 36P5 hydrolase mutant with an N-terminal His_6_-tag and C-terminal GFP (His_6_-Hydrolase-GFP). To immobilize the hydrolase fusion protein, raw cell extract was incubated with Ni-NTA material and washed with PBS buffer. Binding was clearly visible by the color change of the column matrix from blue (Ni-NTA) to yellowish green (GFP). Since the hydrolase was located between His-tag and GFP, the green coloring showed that the intact fusion protein was successfully immobilized (Figure 6A) and retained on the column after repeated use. 

As the high price, as well as the use of heavy-metal ions, prevent large scale applications of Ni-NTA immobilized hydrolase, silica materials were tested as an alternative for immobilization. Various sequences have been described in the literature for protein immobilization on silica material, from short tags to complete proteins [35,36,37,38]. We tested two silica tags: a short tag from the protein CotB1 (C-terminal 14 amino acids) [35] and a longer tag, the ribosomal protein L2 (273 amino acids) [37,39]. CotB1 is a spore coat protein of the soil bacterium *Bacillus cereus* and mediates silica biomineralization which increases acid resistance of the spores [36]. The L2 protein is part of the 50S subunit of the ribosome and is one of the main proteins responsible for rRNA binding. Binding to silica was found experimentally [37]; it is not a natural function but is due to the positive charge of the protein responsible for the binding of negatively-charged rRNA [39]. The GFP and His_6_-tag were retained to allow visual control of immobilization and purification via Ni-NTA, if necessary. 

The CotB1-tag severely impaired expression and folding, indicated by low GFP fluorescence and poor cell growth. Therefore, different arrangements of the fusion protein and different culture conditions were tested to optimize expression. The best result was obtained for the fusion protein His_6_-GFP-Hydrolase-Silica_CotB1_, but due to low yield, it was not followed further, and L2 was tested as an alternative silica tag. Despite its size, the L2-tag did not impair cell growth or protein expression, and thus, further experiments for immobilization on silica material were performed with the fusion construct His_6_-Silica_L2_-Hydrolase-GFP. Analogous to immobilization via His_6_-tag, the cell extract with the expressed fusion protein was directly incubated with two different silica materials, silicic acid (Carl Roth, Karlsruhe, Germany) or silica gel (Sigma-Aldrich, St. Louis, MI, USA) and then washed. The green color of the otherwise colorless silica material provided visual control of the immobilization (Figure 6B,C). Since sand consists largely of silica in the form of quartz, this was also tested as carrier material, and green coloration was likewise observed (Figure 6D). 

The binding efficiency of the different tags to the carrier materials was analyzed and compared by fluorescence microscopy (Figure 6). The clearly visible green coloring of the particles revealed successful immobilization on all materials. Importantly, all particles from the Ni-NTA material (Figure 6A) and both silica materials (Figure 6B,C) were covered uniformly with the hydrolase-GFP fusion. This was not the case with sand (Figure 6D), which might be due to the fact that lower amounts were used, and that sand is a heterogeneous mixture that does not consist exclusively of silicon dioxide.

### 2.5. Enzymatic Inactivation of Florfenicol in Salt Solutions

**Figure 7:**
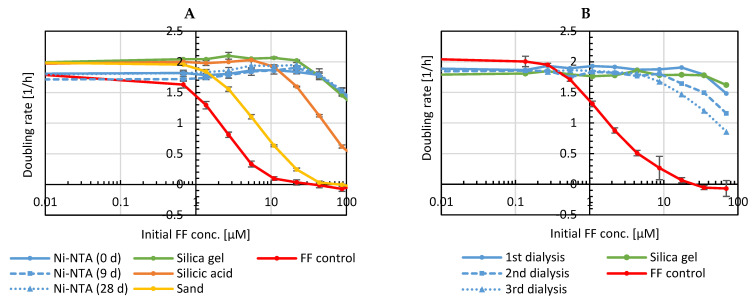
Comparison of florfenicol inactivation in salt solutions. (**A**) Growth assay of BL21 after overnight incubation of FF-containing DYT with immobilized hydrolase 36P5. (*n* = 4 (all Ni-NTA, FF control); *n* = 2 (silica gel 60, silicic acid, sand), time in days for Ni-NTA describes the storage and repeated use); (**B**) Growth test of BL21 after overnight incubation of FF-containing saltwater with hydrolase encapsulated with a semi-permeable membrane after first, second or third use (1st dialysis, 2nd dialysis, 3rd dialysis) or immobilized to silica material (*n* = 4 (FF control), *n* = 1 (dialysis; Silica)).

Enzymatic activities of the hydrolases immobilized on the different materials were analyzed by overnight incubation of the respective materials (approx. 1 mL Ni-NTA or silica slurry) with 10 mL DYT medium containing 700 µM florfenicol. The treated samples were then diluted in DYT medium, and growth assays with sensitive *E. coli* cells in each sample of the dilution series were used as a proxy to estimate the remaining florfenicol. Figure 7A shows obtained cell doubling rates versus initial florfenicol concentration (i.e., calculated theoretical concentration before the enzymatic treatment) of the respective dilution. Hydrolase immobilized on silica, silicic acid and Ni-NTA effectively inactivated florfenicol. Considering the shift of the curve after treatment, the reduction of florfenicol can be estimated. For hydrolase immobilized to Ni-NTA and silica, about 100-fold reduction of florfenicol was achieved, for silicic acid about 25-fold and for sand about 3-fold. The hydrolase immobilized on sand also inactivated florfenicol, albeit to a lesser extent. The lower efficiency is possibly due to the lower amounts of cell extract and sand that were used as well as the material mixture present in the sand as indicated by the inhomogeneous immobilization compared to the other silica materials (Figure 7A).

Recycling of the immobilized hydrolase was verified by repeating the experiment with the Ni-NTA material after 9 and 28 days (Figure 7A). Even after four weeks and repeated use, the hydrolase was still active and cleaved florfenicol as efficiently as directly after immobilization. Robustness of the silica-bound hydrolase was also tested in an almost identical experiment in florfenicol-containing saltwater (3.5% *w*/*v*) NaCl in 10 mM potassium phosphate buffer, pH 8.0), simulating conditions of aquaculture. Comparable inactivation was observed (Figure 7B). Furthermore, florfenicol inactivation in saltwater was also achieved using a hydrolase-containing cell lysate encapsulated with a semi-permeable membrane (10 kDa MWCO dialysis tubing) instead of the immobilized material, and repeated usage was possible as well (Figure 7B).

### 2.6. Enzymatic Inactivation of Florfenicol in Milk

**Figure 8:**
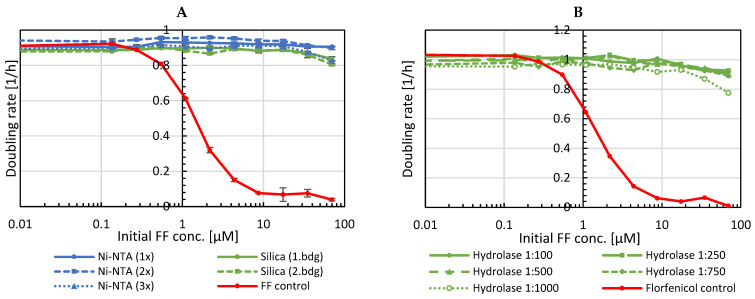
Comparison of florfenicol inactivation in milk. Growth assays in milk of BL21 expressing mCherry after overnight incubation of FF-containing milk (**A**) with hydrolase immobilized on Ni-NTA or silica or (**B**) after addition of crude cell extract with hydrolase.

Besides aquaculture, florfenicol is also widely used in agricultural animal farming. Treatment of dairy cattle, for example, results in antibiotic residues in milk [17,18,19,20,21]. Inactivation of florfenicol is, therefore, of great interest as this would allow safely using this waste milk for calf rearing without risking the development of resistant gut microbes. Hence, it was investigated whether our optimized hydrolase 36P5 can also inactivate florfenicol in milk. Florfenicol-containing milk (250 µg/mL; 700 µM) was incubated with hydrolase immobilized on either Ni-NTA or silica (Figure 8A), respectively, by gentle agitation overnight at 4 °C. As growth tests based on turbidity measurement are not possible in milk, sensitive *E. coli* expressing the red fluorescent protein mCherry as a reporter were used and the fluorescence intensity of the reporter was measured to estimate doubling rates. The hydrolase was active and stable in milk as shown by three consecutive applications of immobilized His_6_-Hydrolase. 

When adding 500 mg dry silica material to cell extract from 1 L *E. coli* culture not all hydrolase was immobilized, and a second capture with 1 g of dry silica material was possible without any performance loss (Figure 8A, Silica (2. bdg)).

In another experiment, the cell extract of *E. coli* expressing a hydrolase-GFP fusion was added directly to florfenicol-containing milk (Figure 8B). In this case, 1 µL crude cell extract was sufficient to inactivate 250 µg florfenicol. Importantly, hydrolase stored at 4 °C for one year was still active (data not shown), indicating that the hydrolase is very robust and prolonged storage is possible.

## 3. Discussion

Pollution of our environment with various waste products poses an increasing threat. Especially the massive use of antibiotics leaves traces in the environment and promotes an alarming rise of antibiotic resistances. Consequently, next to limiting antibiotic use, new strategies are urgently needed for the inactivation of antibiotic residuals. We have devised such a strategy by optimizing a hydrolase enzyme for florfenicol cleavage and applying it in different settings imitating saltwater or waste milk treatment. 

The antibiotic florfenicol is widely used as a veterinary drug but also as a growth promoter in animal feeds [6,40]. Problematic is the high stability of florfenicol under many conditions in various environments. It is hydrolytically stable [41,42] and also virtually resistant to solar photodegradation [43,44], an important degradation mechanism for many other environmental organic pollutants [45]. Consequently, florfenicol has been detected not only in environmental samples [46,47] but also in drinking water [48] and on market products [49,50]. Alarmingly, florfenicol remained stable even after food preparation, such as grilling or cooking, and its amount was only decreased by its transfer from the meat to boiling juice during the cooking process [51]. As a result, the emergence of florfenicol resistance has dramatically increased not only among different bacteria collected from farm animals [5,52,53,54] or from environmental samples from such farms [55], but also from bacteria found in drinking water [56], market food [57] as well as from human samples [23,30,31,40,58]. Importantly, many of these bacteria are multi-drug resistant owing to the fact that florfenicol resistance is mainly attributed to different, often not very specific resistance mechanisms, such as efflux pumps (e.g., *floR*, *fexA/B*, *pexA/B*, *AcrAB-TolC*) [24,59], 23S rRNA methyltransferase (*cfr*), which prevents the binding of florfenicol to the ribosome [26], or members of the ARE ABC-F proteins (antibiotic resistance ATP-binding cassette superfamily F lineage) (*optrA*, *poxtA*), which protect bacterial ribosomes from antibiotic-mediated inhibition [25,27]. The Cfr rRNA methyltransferase, for example, methylates A2503 of 23S rRNA close to the peptidyl transferase center and confers resistance to five different classes of antimicrobial agents [26].

So far, florfenicol degradation has been demonstrated using UV irradiation from a 300 W high-pressure mercury lamp (λ ~ 253.7 nm) alone or in the presence of H_2_O_2_ or FeSO_4_ [60]. Similarly, UV in combination with sodium persulfate was used in a collimated-beam bench reactor equipped with a UV lamp (UV-C, 75 W) and a magnetic stirrer [61]. However, we believe that for efficient, large-scale, and potentially turbid solutions, enzymatic degradation provides a more feasible, less energy consuming and environmentally friendlier solution. However, contrary to chloramphenicol and thiamphenicol, which can also be inactivated enzymatically by acetylation via different types of chloramphenicol acetyltransferases (CAT) [22], florfenicol is resistant to this reaction (see also Figure 1) due to the fluor residue at C-3 instead of the hydroxyl group which serves as acceptor site for the acetylation. 

EstDL136 is the only enzyme reported to be able to inactivate florfenicol by hydrolysis. However, since the enzyme was found in a function-based screening searching for lipolytic activity in a metagenome library prepared from alluvial soil collected from Eulsukdo island (Saha-Gu, Busan, Korea) [28], it seems likely that cleavage of florfenicol and chloramphenicol is not the main reaction and is only a result of broad substrate specificity. Indeed, the selection occurred based on tributyrin hydrolysis, and two clones carrying EstDL26 and EstDL136 were selected for further analysis based on the observation that they possessed chloramphenicol acetate esterase (CAE) activity counteracting CAT which served as a selection marker for plasmid maintenance [28]. Only a later study from the same group reported that EstDL136 does not only reactivate chloramphenicol by deacetylation of Cm acetates but also shows a promiscuous amidase activity leading to hydrolysis of chloramphenicol as well as florfenicol [29]. 

Using error-prone PCR to randomly introduce new mutations in the hydrolase gene in combination with metabolic selection with florfenicol, which couples desired enzyme properties to cell growth, we enriched clones with improved hydrolase activity. For selection, first, the *E. coli* expression strain RV308 was used, which is widely utilized in industry [62,63]. During metabolic selection an additional acquired resistance was observed, which occurred independently of the hydrolase. Thus, for further experiments the *E. coli* strain BL21 was used, which did not show such adaptations and was, therefore, better suited for selection. The hydrolase mutant 36P5 conferred a significant increase in the *E. coli* doubling rate under selective pressure. Importantly, the increased resistance compared to the wild-type enzyme was exclusive for florfenicol (Figure 4) confirming selection for the desired activity. Metabolic selection has the advantage that it selects simultaneously also for good expression, folding, solubility and stability, all important traits for industrial use but very difficult to predict rationally [64,65]. Indeed, our selected hydrolase variant 36P5 was active in all media tested (DYT, saltwater, milk) and showed good performance at different temperatures (37 °C, room temperature, 4 °C).

While enzymatic inactivation of antibiotics could serve as a general route for decontamination, not every enzyme is suitable for such an application. Chloramphenicol-acetyl transferase, for example, inactivates chloramphenicol by acetylation of two hydroxy groups and requires acetyl-CoA as co-substrate in stoichiometric amounts. As the cellular concentration of acetyl-CoA is low [66,67,68], using cell lysates or purified enzymes requires the supplementation with acetyl-CoA (Figure S3) or possibly another form of an activated acetyl group. Furthermore, hydrolysis of 1,3-diacetylchloramphenicol by CAT [69] or other mechanisms [28,70] could reactivate the antibiotic. In contrast, our optimized EstDL136 hydrolase mutant 36P5 does not have any of these restrictions. It only requires water as co-substrate and can thus function in virtually every aqueous solution as demonstrated with milk and saltwater. Furthermore, it inactivates florfenicol by cleavage making the reverse reaction highly unlikely in dilute solutions and is, therefore, highly suitable for an ecofriendly inactivation of florfenicol.

Industrial use of enzymes is manifold and, depending on production costs, recycling of enzymes for repeated use can be desirable. Consequently, different strategies to apply enzymes in an active but insoluble form, which enables easy recovery from a process, have been developed, such as carrier-bound immobilization through physical or chemical attachment (physisorption, chemisorption), enzyme entrapment through encapsulation and formation of cross-linked enzyme aggregates (CLEA) [71]. Limitations are mainly associated with a loss in activity.

We have tested and successfully applied three different reuse approaches, two carrier-bound immobilization strategies using either His_6_-*tag* binding to Ni-NTA derivatized beads or silica_L2_-*tag* binding to silica material, and as a third strategy, enzyme entrapment through encapsulation in a semi-permeable membrane. Importantly, florfenicol inactivation was achieved in all cases proving that the enzyme remained active. The His_6_-tag is widely used as a purification tag because of its small size and minimal disturbance of protein function in most instances [72]. Indeed, the His_6_-tagged hydrolase was well expressed and immobilized, and the material was stable and reusable over an extended time period. However, large scale use is hampered by the high cost of the materials, as well as some enzyme and heavy metal ion leaching [72]. Therefore, we also tested silica materials as very cost-efficient and highly abundant materials. Several silica-tags are described in the literature [35,36,37,38], however, we found that some severely reduced recombinant enzyme expression yield as seen by a lack or very low amount of green fluorescence in our fusion proteins. In our hands, the L2 protein performed best, and immobilization was achieved to different silica materials. As it had been reported that even naturally occurring volcanic ash (Shirasu from Mount Sakurajima in Kagoshima, Japan) with about 70% silica content could be used to bind silica-tagged proteins [36], we also tested ordinary sand and observed immobilization as well, albeit with lower efficiency, which is probably due to a lower silica content.

Enzyme immobilization by entrapment retains the enzyme in carriers with different porosity and permeability, often achieved by hydrogels, polymers or nanomaterials. As a simple entrapment strategy, we used a dialysis tube with a molecular weight cutoff of 10 kDa. Inactivation of florfenicol in saltwater was efficiently and repeatedly achieved using the same encapsulated enzyme material. Inactivation of florfenicol in milk was also possible but was considerably slower (Figure S4), probably due to clogging of the pores, indicating the importance of pore size for efficient exchange of substrate and products but retaining the enzyme to prevent leaching. For many entrapment methods, material thickness and shape can be controlled to optimize mass transport of substrate and product [73].

Inactivation of antibiotics can not only help to prevent the emergence of resistances but can also aid in the safe use of products, such as waste milk, which is produced when dairy cows are treated with antibiotics. This waste milk, which constitutes approximately 1% of the milk produced in the European Union [19], contains substantial amounts of antibiotic residuals and is commonly fed to calves promoting the emergence of antibiotic-resistant bacteria in their intestinal and respiratory tracts [18,19,20,21]. A recent report from the European Food Safety Agency (EFSA) on the “Risk for the development of antimicrobial resistance (AMR) due to feeding of calves with milk containing residues of antibiotics” discusses problems associated with practicability and compliance when prohibiting the use of certain waste milk. They propose antibiotic inactivation as an alternative counter-measure and list some strategies for the inactivation of β-lactam antibiotics including heat treatment, pH increase, electrochemical oxidation, fermentation and enzymatic treatment [19]. Similarly, Kitazono et al. propose an electrochemical process to remove antibiotics from waste milk [74]. 

Consequently, we believe that the presented work provides valuable new strategies for florfenicol inactivation by an optimized hydrolase in soluble or immobilized forms. Such a process would, for example, provide a gentle and environmentally-friendly treatment option for waste milk, which could then safely be fed to calves without the risk of promoting AMR.

## 4. Materials and Methods

### 4.1. Plasmids

The plasmid pUEst136 containing the hydrolase gene (EstDL136) was a kind gift from Prof. Dr. Seon-Woo Lee at Dong-A University in Busan, Republic of Korea. The hydrolase gene was amplified by PCR using the primers pf_Cm-Hydrolase_XbaI (GCAAGGTCTAGATGCCGTTAAACCCCCATG) and pr_Cm-Hydrolse_PstI (CACGGTCTGCAGAGCGAGGTCTCTTTTAAG) and cloned via XbaI and PstI into pBAD-Kan, a derivative of pBAD/His (Life Technologies, Carlsbad, CA, USA) with a kanamycin resistance cassette instead of an ampicillin resistance cassette. The resulting plasmid, pBAD-Hydrolase-His_6_, was used for growth assays, library generation and protein expression for NMR experiments. For immobilization, the best hydrolase mutant 36P5 was PCR amplified using the primers pf_Hydrolase_AN (CATGCTAGCCCGTTAAACCCCCATGTCGAAGC) and pr_Hydrolase_AN_wo_Stop (AGGCGCGCCAGCGAGGTCTCTTTTAAGATTGGATGCAC) and cloned via NheI and AscI into the pAR2000-His_6_-GFP vector (ampicillin resistance), a derivative of the pQE16 plasmid (Qiagen, Hilden, Germany) with the addition of a GFP gene in frame with the His_6_ gene and the addition of the lac repressor gene, to yield pAR2000-His_6_-Hydrolase-GFP. The Silica L2 sequence was obtained by amplifying the ribosomal rplB gene from BL21 using the primers pf_rplB_SLiCE (TGCACCATCACCATCACCATACCGGTGCAGTTGTTAAATGTAAACCGACATCTCCGG) and pr_rplB_SLiCE (CGACATGGGGGTTTAACGGGCTAGCTTTGCTACGGCGACGTACGATGAATTTATCAG) and cloned using the SLICE protocol [75] into pAR2000-His_6_-Hydrolase-GFP linearized with NheI. 

### 4.2. Strains

All experiments were peformed using the *E. coli* strain BL21 gold (F^–^ *ompT hsdS*(*r_B_*^–^ *m_B_*^–^) *dcm*^+^ Tet*^r^ gal endA*; Agilent, Santa Clara, CA, USA). Initial experiments were performed with the *E. coli* strain RV308 (l*acI*^q−^, su^−^, *ΔlacX74*, *gal*, IS II::OP308, *strA*) [63], however, this strain proved to be unsuited for library selection experiments as it was able to adapt to florfenicol pressure (Figure S2).

### 4.3. Library Generation and Selection

Hydrolase libraries were generated by random mutagenesis with the Genemorph II Random Mutagenesis Kit (Stratagene, La Jolla, CA, USA) according to the manufacturer’s instructions with the primers pf_Cm-Hydrolase_Shuffling (CTAGAAATAATTTTGTTTAACTTTAAGAAGGAGTCTAGATG) and pr_Cm-Hydrolase_Shuffling (TGATGATGACCGGGCTGCAG) and using 500 ng template DNA for a low to medium mutation rate. The error-prone PCR product was digested and ligated into pBAD-Kan, dialyzed against water and electroporated into electrocompetent *E. coli* BL21 gold. Library size was 8 × 10^6^. Cells were pooled in DYT and used to inoculate 50 mL DYT, taking care to achieve at least 20-fold coverage of the library, containing 50 µg/mL kanamycin and 0.02% (*w*/*v*) arabinose for hydrolase expression to prime the system for the following selection. After 4 h of incubation at 37 °C, this culture was used to inoculate the selection culture of 20 mL DYT supplemented with 0.02% arabinose and 3 µg/mL (8.7 µM) FF to an OD_550_ of 0.1. After incubation overnight at 37 °C, the culture was split and a second passage was performed in a similar way for 24 h in media supplemented with arabinose and 3 µg/mL or 6 µg/mL FF, respectively, yielding culture FF3-3 or FF3-6, respectively. Glycerol stocks were prepared from each step. While we found that RV308 can adapt to some florfenicol pressure, we did not observe this behavior in BL21. However, to ensure that the bacteria did not show any adaptation to the selection pressure independent of the hydrolase activity, plasmids were purified from the FF3-3 and FF3-6 pool and transformed into fresh BL21 gold. Cells were pooled and hydrolase expression was induced in the same manner as for the initial library. Cells were then plated on agar plates containing 0.02% arabinose and 3, 4, 5, 6 and 7 µg/mL FF and incubated at 37 °C for 2 days. Clones were picked and plasmids were sequenced (33P5, 36P4, 36P5, 36P6, 36P7) and also transformed in fresh BL21 gold to prevent any unwanted adaptation to Florfenicol.

### 4.4. Bacterial Growth Assays

To determine the doubling rates, 1:200 to 1:500 dilutions with DYT or milk of BL21 overnight cultures were grown to early log phase for approx. 2 h (pre-culture) and then 100 µL were dispensed in 96-well microtiter plates already filled with 100 µL of medium supplemented with decreasing antibiotic concentrations (“log2”-dilution series) and as a control without antibiotic. Cultures were orbitally shaken at 37 °C in a plate reader (Infinite M1000 pro, Tecan, Männedorf, Switzerland) and the optical density at 550 nm (OD_550_) as a measure of cell density was recorded every 5 min for 24–48 h. The blank value (medium without cells) was subtracted before calculating the log_2_ doubling rate µ for the respective antibiotic concentration from the exponential growth phase, i.e., the linear part of the log_2_ *OD_t_* vs time plot ($\log_{2}OD_{t}=µ\;t+\log_{2}OD_{0}$). 

Inhibition zone assays were performed by plating dense expression cultures of BL21 harboring different hydrolase mutants and placing a filter plate soaked with 5 µL of 25 mg/mL florfenicol solution after drying onto the center of the plate. After overnight incubation at 37 °C, the size of the inhibition zone was measured from the scanned plates using the program ImageJ [76] and calculated as a percentage deviation from the wild-type hydrolase. To minimize measurement errors, three independent measurements were taken, and the mean and standard deviation was calculated.

### 4.5. Protein Expression

For larger-scale expressions for NMR and immobilization experiments, 1 l DYT was inoculated with 10 mL overnight culture and incubated at 30 °C. At an OD_550_ of approx. 0.6, expression was induced with 0.02% (*w*/*v*) arabinose or 1 mM IPTG, depending on the plasmid. The culture was grown overnight at 30 °C (or 26 °C for silica tag). Cells were then pelleted by centrifugation (5000× *g*, 30 min, 4 °C), resuspended in PBS (10 mM Na_2_HPO_4_, 1.8 mM KH_2_PO_4_, 137 mM NaCl, 2.7 mM KCl, pH 7.4; for Ni-NTA immobilization) or Tris-buffer (25 mM Tris-HCl, pH 8.0; for silica immobilization) and lysed by sonification (Digital Sonifier 250, Branson Ultrasonic). Cell debris was removed by centrifugation (20,000× *g*, 30 min, 4 °C), the supernatant was filtered (0.45 µm filter) and used for protein purification, or directly for immobilization and florfenicol removal in milk.

### 4.6. Protein Purification

For NMR measurements, the His-tagged hydrolase was purified from the crude cell extract by immobilized metal-ion affinity chromatography (IMAC) using a 1 mL Ni-NTA agarose (Qiagen) column. The column was washed twice with 10 column volumes PBS/10 mM imidazole and the enzyme was competitively eluted with 2 × 2.5 mL PBS/250 mM imidazole. Further purification of the hydrolase was achieved by size exclusion chromatography (SEC) in PBS using a Superdex75 column (10/300 GL, Cytiva, Marlborough, MA, USA) controlled by an automated liquid chromatography system (ÄKTA Purifier 10, Cytiva). Finally, samples of the fractions were analyzed by SDS-PAGE and Coomassie staining.

### 4.7. NMR Measurements

Hydrolase cleavage was followed continuously by recording ^1^H-NMR spectra in 4 min intervals of a solution containing 700 µM florfenicol in PBS mixed with 0.25 µM hydrolase (EstDL136 or 36P5 mutant) using a 600 MHz NMR spectrometer (Avance III, Bruker, Billerica, MA, USA) equipped with 5 mm room temperature probe with z-gradients. Each ^1^H-NMR measurement contained 64 scans. Acquisition time and recycling delay were set to 2 s and 1.5 s, respectively. Bruker TopSpin v3 was used for spectra processing and visualization.

### 4.8. Immobilization

Immobilization on Ni-NTA carrier was achieved by incubating 10 mL crude cell extract (in PBS for immobilization on Ni-NTA, or 25 mM Tris, pH 8.0 for immobilization on Silica) of *E. coli* expressing the 36P5 hydrolase mutant as His_6_-hydrolase36P5-GFP or His_6_-silica_L2_-hydrolase36P5-GFP fusion with 1 mL Ni-NTA agarose slurry in PBS or 200–500 mg Silica gel 60 (40–63 µm, Sigma-Aldrich) or silicic acid (No. 0201.1, Carl Roth, Karlsruhe, Germany) suspended in 25 mM Tris, pH 8.0. For immobilization on sand, a spatula tip of ground sand was incubated with 1 mL cell extract in Tris-buffer. Materials were washed several times with PBS (Ni-NTA) or 25 mM Tris (Silica) before and after use. Materials were stored at 4 °C in the respective buffer supplemented with 200 µg/mL ampicillin and 100 µg/mL kanamycin to prevent microbial growth.

### 4.9. Florfenicol Inactivation Assay

To examine florfenicol cleavage in salt solutions, about 1 mL of the immobilized hydrolase slurries were mixed with either 10 mL DYT medium or 10 mL saltwater (3.5% (*w*/*v*) NaCl, 10 mM Potassium-Phosphate, pH 8.0) each supplemented with florfenicol (250 µg/mL or 700 µM) and incubated overnight (approx. 16 h) at room temperature with slow agitation. The solutions were then decanted and sterile filtered (0.22 µm). The remaining florfenicol content in the treated solutions was semi-quantitatively determined using bacterial growth assays as a proxy. Treated solutions were dispensed 100 µL each at a log2 dilution in a microtiter plate. To each dilution 100 µL FF-sensitive BL21 were added, and growth curves were recorded identical to the bacterial growth assays mentioned above. Doubling rates were calculated from the initial exponential growth phase and plotted against the theoretical florfenicol concentration without enzymatic treatment. 

The protocol was adapted for inactivation in milk. To keep milk fresh, enzymatic inactivation was performed at 4 °C. As the turbidity in milk prevents growth measurements by optical density, BL21 harboring a plasmid expressing the red fluorescent protein mCherry was used combined with fluorescence measurements for growth assays. Since milk cannot be easily sterile filtered, ampicillin was added to prevent other microbial growth and maintain the mCherry plasmid. For the growth assay, BL21/mCherry was grown in milk and analogous to the OD measurement, the fluorescence emission intensity (excitation 587 nm, emission 610 nm) was measured as a proxy of cell growth from which doubling rates were deducted.

Similar to the inactivation with immobilized hydrolase, crude cell extract containing approximately 10 µM His_6_-Hydrolase-GFP (as judged by GFP absorbance measured at 488 nm where crude extracts have a low background) was used for florfenicol inactivation in milk or saltwater containing 700 µM florfenicol. The crude cell extract was used either directly in dilutions ranging from 1:100 to 1:1000, or 4 mL crude cell extract in a 10 kDa molecular weight cut-off (MWCO) dialysis tubing was added to 40 mL salt water or milk supplemented with 700 µM. Incubations and growth tests were carried out as described above.

## Figures and Tables

**Table 1:** Overview of the amino acid mutations found in the selected hydrolase variants. Listed are the amino acids and the corresponding codons in parentheses.

Amino Acid Position ^1^	Location in the Protein ^2^	Wt Hydrolase	Mutant 33P5 (1×) ^3^	Mutant 36P4 (2×)	Mutant 36P5 (2×)
51	surface	Asn (AAT)			Asp (GAT)
186	near Cm	Val (GTA)	Ala (GCA)	Ala (GCA)	Ala (GCA)
189	surface	Asn (AAT)		Ile (ATT)	
211	near Cm	Met (ATG)	Leu (TTG)		Leu (TTG)
226	surface	Asn (AAT)			Asp (GAT)
286	near Cm	Ser (AGC)		Gly (GGC)	

^1^ Numbering according to UniProt G3CR02. ^2^ The location of the mutated amino acids in the protein was determined based on the three-dimensional structure of hydrolase in complex with chloramphenicol (PDB code: 6IEY) [33]. ^3^ Number of occurrences.

## Supplementary Materials

The following are available online at https://www.mdpi.com/article/10.3390/antibiotics11040443/s1; Figure S1: Comparison of selected hydrolase mutants; Figure S2: Adaptation of *E. coli* RV308 to florfenicol pressure; Figure S3: Comparison of CAT and hydrolase EstDL136 for Cm inactivation; Figure S4: Florfenicol inactivation in milk using encapsulated hydrolase 36P5; gene sequences for constructs used.
