# Supplementary material for: Strategies for Enzymatic Inactivation of the Veterinary Antibiotic Florfenicol
Source: Antibiotics (Basel). 2022 Mar 25;11(4):443. doi: 10.3390/antibiotics11040443 (PMC9029715; doi:10.3390/antibiotics11040443)
Supplement: Supplementary file 1 [file antibiotics-11-00443-s001.zip › antibiotics-1649436-supplementary.pdf]

antibiotics

MDPI

Supplementary Materials

Strategies for Enzymatic Inactivation of the Veterinary Antibiotic Florfenicol

Marik M. Müller [1], Ruslan Nedielkov [2] and Katja M. Arndt [1,*]

[1] Molecular Biotechnology, University of Potsdam, Karl-Liebknecht-Str. 24-25, 14476 Potsdam, Germany
[2] Institute of Chemistry, University of Potsdam, Karl-Liebknecht-Str. 24-25, 14476 Potsdam, Germany
* Correspondence: katja.arndt@uni-potsdam.de

1. Comparison of selected hydrolase mutants

Mutants obtained after selection were compared in growth assays using BL21 expressing different hydrolase variants (Figure S1). Growth tests at different florfenicol concentrations revealed a significant improvement of all selected mutants over the wild-type hydrolase EstDL136. Mutants 33P5 and 36P5 showed similar performance, and mutant 36P5 was chosen for further analysis as this mutant was found twice.

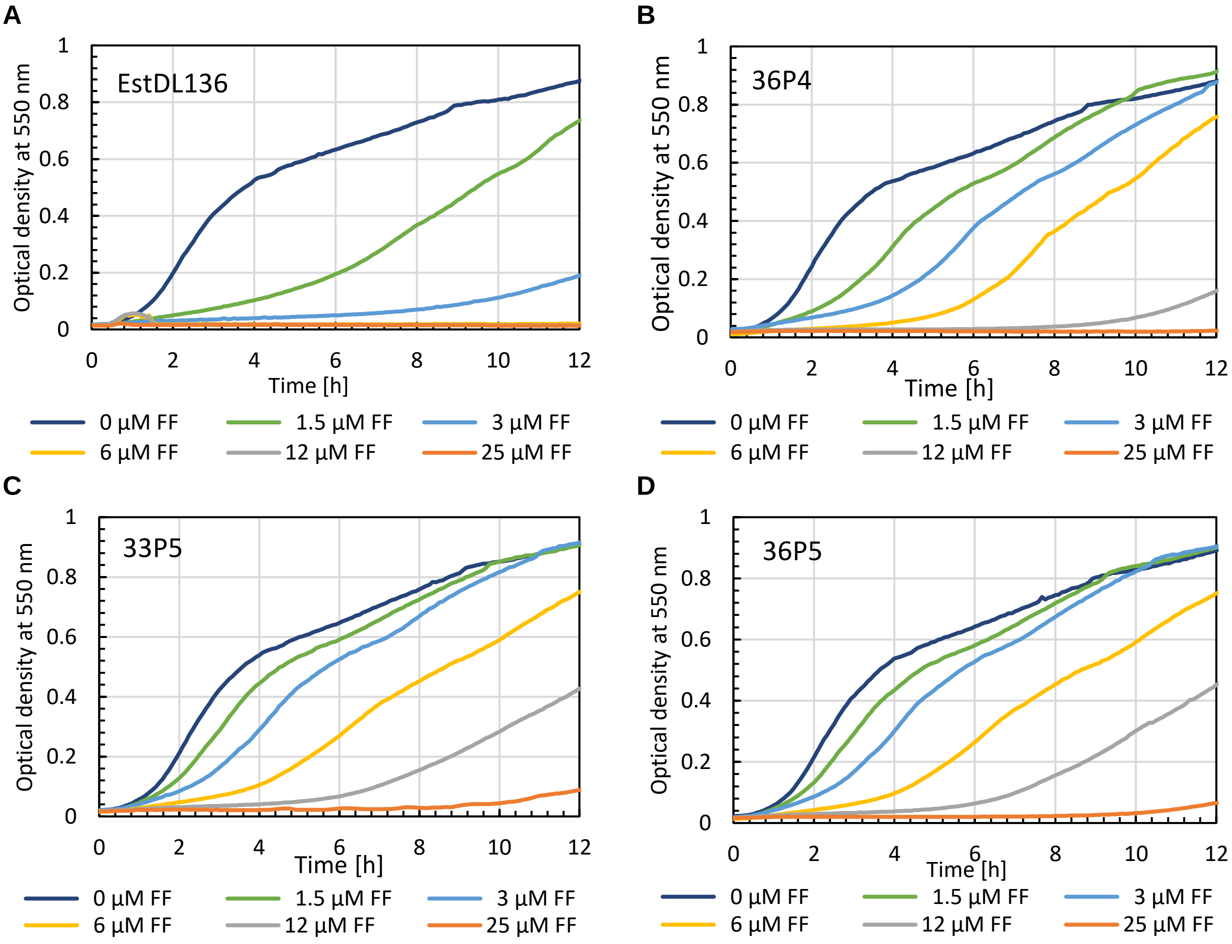

Figure S1. Comparison of selected hydrolase mutants. Growth curves at different florfenicol concentrations of BL21 expressing **(A)** wild-type hydrolase EstDL136, (**B**) mutant 36P4, (**C**) mutant 33P5, or (**D**) 36P5.

2. Adaptation of *E. coli* RV308 to florfenicol pressure

Optimization of the hydrolase enzyme was first attempted in the *E. coli* expression strain RV308, which is widely utilized in industry [62,63]. Enriched hydrolase variants showed a very heterogeneous pattern of mutations including some truncations but similar improvement in florfenicol tolerance. Doubling rates at different florfenicol concentrations of RV308 expressing the wild-type hydrolase EstDL136 were compared to two of the selected hydrolase mutants (M1 and M2), which were obtained after selection with increasing florfenicol pressure up to 35 µM (Fig. S1A, solid lines).

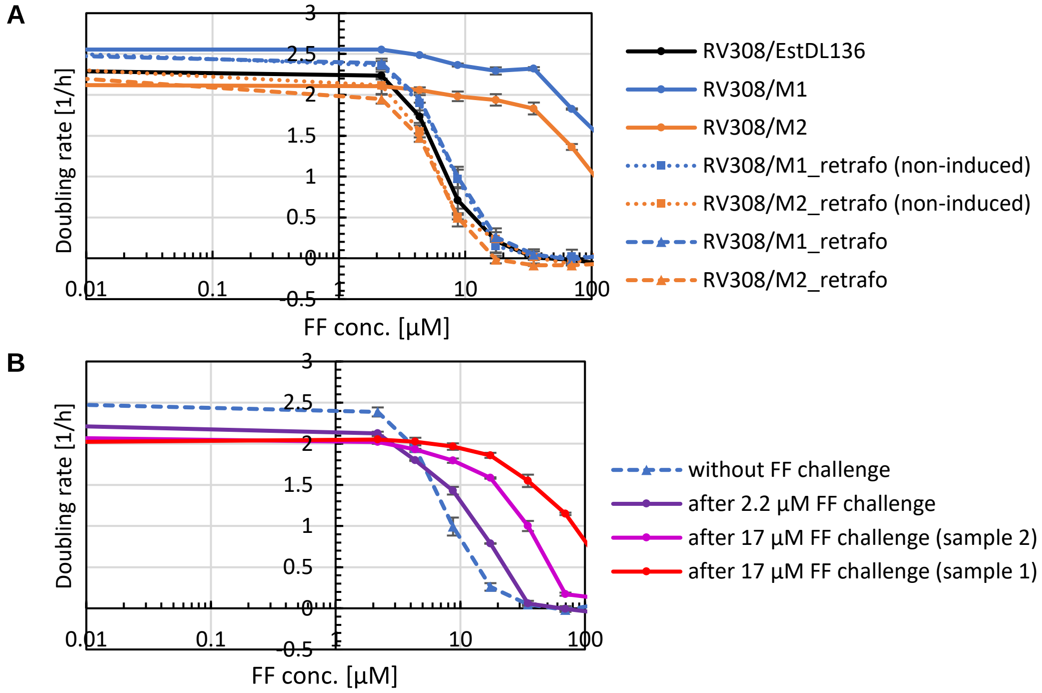

Figure S2. Adaptation of *E. coli* RV308 to florfenicol pressure. (A) Growth assay of *E. coli* RV308 expressing wild-type hydrolase EstDL136 (black solid line; n=4) or mutants M1 or M2 obtained after metabolic selection (solid line: RV308/M1, n=2; RV308/M2, n=4), compared to fresh RV308 retransformed with the plasmid for M1 or M2 without induction (dotted line: RV308/M1_retrafo (non-induced), n=4; RV308/M2_retrafo (non-induced), n=2) and with induction (dashed line: RV308/M1_retrafo, n=6; RV308/M2_retrafo, n=2). (**B**) Growth assay of RV308 expressing hydrolase mutant M1 at different FF concentration directly after retransformation without prior FF challenge (blue dashed line; n=2) or after overnight challenge with 2.2. or 17 µM FF (solid line; n=2). Two independent samples were prepared for the 17 µM FF challenge (sample 1 and 2).

To examine whether the increase in florfenicol resistance was caused by the mutated hydrolase, plasmids were isolated, transformed into fresh *E. coli* RV308 cells, and the growth assay was repeated with (dashed line, Fig. S1A) or without the inducer arabinose (dotted line, Fig. S1A). After isolation and retransformation of the plasmid, the selected mutants did not show any improvement of the wild-type hydrolase. Furthermore, there was no effect seen whether expression of the hydrolase had been induced or not. This observation suggests that pre-treatment of the cells might have a significantly larger influence on cell growth than any hydrolase variant.

This assumption was confirmed by growth assays of the retransformed mutant M1 either without prior florfenicol challenge or after preincubation overnight with 2.2. µM or 17 µM florfenicol (Fig. S1B). A clear shift towards higher florfenicol tolerance was observed after prolonged contact with florfenicol, probably due to some metabolic

adaptation. The preincubation with 17 µM florfenicol was done independently for two samples (Fig. S1B, sample 1 and 2), and measurements suggest that either the rate or the extent of the adaptation might differ in individual cells. A similar experiment was performed with RV308 without any hydrolase, and a similar adaptation was observed. In general, the ability of RV308 to adapt increased florfenicol concentrations independent of the presence of the hydrolase renders this *E. coli* strain RV308 is not suitable for selecting optimized hydrolase mutants.

3. Comparison of CAT and hydrolase EstDL136 for Chloramphenicol inactivation

The enzyme CAT is a well-established resistance marker and efficiently inactivates chloramphenicol inside the cell (Figure 1). However, the use of CAT for industrial inactivation of antibiotics is hampered by the stochiometric use of the cofactor Acetyl-CoA. This has been demonstrated in an assay, where samples of chloramphenicol-containing DYT medium (25 µg/ml Cm) were incubated with decreasing amounts from a cell extract (2 ml) prepared from a 20 ml *E. coli* BL21 culture expressing either CAT or EstDL136. For comparison, incubation with the CAT-containing extract was done without or with addition of Acetyl-CoA. After overnight incubation, growth of sensitive BL21 was used as proxy for remaining Cm, and growth rates were calculated relative to growth in medium without Cm (Figure S2). As the cellular concentration of acetyl-CoA, CAT requires supplementation with acetyl-CoA, whereas the hydrolase EstDL136 only needs water for inactivation of Cm or FF.

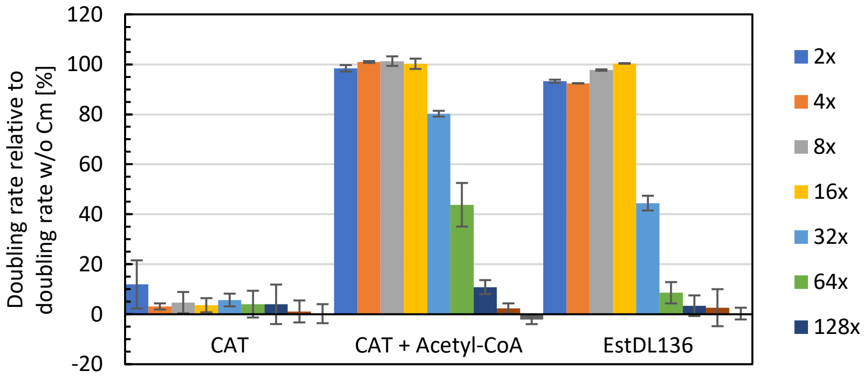

Figure S3. Comparison of CAT and hydrolase EstDL136 for Cm inactivation. Growth assay of *BL21* after incubation of Cm-containing DYT with cell extract containing hydrolase EstDL136 or CAT with or without addition of Acetyl-CoA. Growth rates were calculated as percentage from growth rates obtained in medium without Cm (n=5 (CAT); n=2 (EstDL136, CAT+Actyl-CoA)). The legend shows the dilution factor of the cell extract.

4. Florfenicol inactivation in milk using encapsulated hydrolase

Similar to florfenicol inactivation in saltwater using encapsulated hydrolase, the same experiment was repeated in florfenicol-containing milk (Figure S3). Florfenicol inactivation was possible but considerably slower compared to saltwater (Figure 7B). Since the hydrolase showed good activity in both media as seen for florfenicol inactivation with immobilized hydrolase (Figure 7A and Figure 8A), the lower efficiency using the encapsulated hydrolase is most likely caused by clogging of the pores of the dialysis tube with milk components.

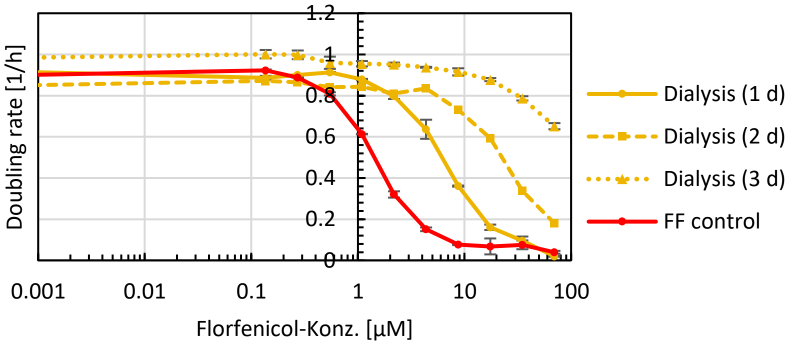

Figure S4. Florfenicol inactivation in milk using encapsulated hydrolase 36P5. Growth assay of *BL21* after incubation of FF-containing milk with hydrolase encapsulated with a semi-permeable membrane, measured after 1, 2 and 3 days (n=2 (FF control, dialysis (1d), dialysis (3d); n=1 (dialysis (2d)).

5. Sequences of constructs used in this study

5.1 Gene encoding Hydrolase-His6 in pBAD-Kan

The gene for the wild-type hydrolase EstDL136 is shown in red, and the gene for the His-tag in blue. Black and blue parts are part of the pBAD-Kan vector. Restriction sites are underlined.

TCTAGATGCCGTTAAACCCCCATGTCGAAGCCCTGTTGCAAATGATGGCACAAATGCCCGCACCCGAT
TTCTCTGTAGCAAATCCGGCTGAAATCCGAGCGGTCTTTGACAATCCAATGCCGCTGGCCGCGCCGCC
TCAGGTTGCCCGGGTAGAGAATATTGCGATTTCCCTCGACGGGCGGGATCTTGATGCGCGGCTTTACGT
GCCCGAAGACGCGGATGAGCGACCTGCACTGATGGTCTATTATCATGGCGGCGGTTGGGTCATCGGA
ACTTTGGATACCCACGACGGCACCTGCCGTGCTCTAGCGCAAAAAAGTGGCTGCGCTGTTTTGTCGAT
CGCTTATCGGCTGGCACCGGAGTATCGCTATCCGGCGCCCGCCGAAGATTGCTATGACGCGCTGGTCT
GGGCCAAGCAAAATGCCGCAACTCTGGGTGTGGATGGCGATCGCCTTGCCGTTGGCGGTGATAGCGC
AGGCGGCAATCTTGCGGCTGCCGTTGCCATAATGGCCCGCGACCGGAATGGTCCCGCGCTTCGCCATC
AGTTGCTCATTTATCCCGTAACCGACAATGATTTCACTCTTGCCTCCTATGCCGAAAATGGCGGGGGCG
AATATTATCTCAGTACCGATGGCATGCGCTGGTTCTGGGGCCATTATCTTGGCGATACGGCAGCAGAA
AATGCGCCGCTTGCCGCCGTTCTGAATGTGGCGGATCTGTCTGGCCTCGCGCCGGCTACGGTTATCACG
GCGGAATATGACCCCTTGCGTGACGAGGGCATTGCCTATGCCAAAAAGCTTGATGCCGCAGGCGTGC
CGGTTGATGCAGCGACGGCGCCTGGCATGATCCACGGTTTCTTCAGCATGTTCGAAGCTGTGCCCGAT
AGTTGGGAATGGATAGAACGAGGTGCATCCAATCTTAAAAGAGACCTCGCTCTGCAGCCCGGTCATC
ATCACCATCACCATTGA

5.2 Gene encoding His6-Hydrolase36P5-GFP in pAR2000

The gene for the His-tag is shown in blue, the gene for the optimized hydrolase mutant 36P5 is shown in red, the flexible linker in purple, and GFP in green. Restriction sites are underlined.

GCATGCACCATCACCATCACCATGCTAGCCCGTTAAACCCCCATGTCGAAGCCCTGTTGCAAATGATG
GCACAAATGCCCGCACCCGATTTCTCTGTAGCAAATCCGGCTGAAATCCGAGCGGTCTTTGACAATCC
AATGCCGCTGGCCGCGCCGCCTCAGGTTGCCCGGGTAGAGGATATTGCGATTTCCCTCGACGGGCGGG
ATCTTGATGCGCGGCTTTACGTGCCCGAAGACGCGGATGAGCGACCTGCACTGATGGTCTATTATCAT
GGCGGCGGTTGGGTCATCGGAACTTTGGATACCCACGACGGCACCTGCCGTGCTCTAGCGCAAAAAA
GTGGCTGCGCTGTTTTGTCGATCGCTTATCGGCTGGCACCGGAGTATCGCTATCCGGCGCCCGCCGAAG
ATTGCTATGACGCGCTGGTCTGGGCCAAGCAAAATGCCGCAACTCTGGGTGTGGATGGCGATCGCCTT
GCCGTTGGCGGTGATAGCGCAGGCGGCAATCTTGCGGCTGCCGTTGCCATAATGGCCCGCGACCGGA
ATGGTCCCGCGCTTCGCCATCAGTTGCTCATTTATCCCGCAACCGACAATGATTTCACTCTTGCCTCCTA
TGCCGAAAATGGCGGGGGCGAATATTATCTCAGTACCGATGGCTTGCGCTGGTTCTGGGGCCATTATC
TTGGCGATACGGCAGCAGAAGATGCGCCGCTTGCCGCCGTTCTGAATGTGGCGGATCTGTCTGGCCTC
GCGCCGGCCACGGTTATCACGGCGGAATATGACCCCTTGCGTGACGAGGGCATTGCCTATGCCAAAA
AGCTTGATGCCGCAGGCGTGCCGGTTGATGCAGCGACGGCGCCTGGCATGATCCACGGTTTCTTCAGC
ATGTTCGAAGCTGTGCCCGATAGTTGGGAATGGATAGAACGAGGTGCATCCAATCTTAAAAGAGACC
TCGCTGGCGCGCCTTCAGGTAGCTCTGGCACTTCAAGCGGTACTAGTATGGTGAGCAAGGGCGAGGA
GCTGTTCACCGGGGTGGTGCCCATCCTGGTCGAGCTGGACGGCGACGTAAACGGCCACAAGTTCAGC
GTGTCCGGCGAGGGCGAGGGCGATGCCACCTACGGCAAGCTGACCCTGAAGTTCATCTGCACCACCG
GCAAGCTGCCCGTGCCCTGGCCCACCCTCGTGACCACCCTGACCTACGGCGTGCAGTGCTTCAGCCGC
TACCCCGACCACATGAAGCAGCACGACTTCTTCAAGTCCGCCATGCCCGAAGGCTACGTCCAGGAGC
GCACCATCTTCTTCAAGGACGACGGCAACTACAAGACCCGCGCCGAGGTGAAGTTCGAGGGCGACAC
CCTGGTGAACCGCATCGAGCTGAAGGGCATCGACTTCAAGGAGGACGGCAACATCCTGGGGCACAAG
CTGGAGTACAACTACAACAGCCACAACGTCTATATCATGGCCGACAAGCAGAAGAACGGCATCAAG
GTGAACTTCAAGATCCGCCACAACATCGAGGACGGCAGCGTGCAGCTCGCCGACCACTACCAGCAGA
ACACCCCCATCGGCGACGGCCCCGTGCTGCTGCCCGACAACCACTACCTGAGCACCCAGTCCGCCCTG
AGCAAAGACCCCAACGAGAAGCGCGATCACATGGTCCTGCTGGAGTTCGTGACCGCCGCCGGGATCA
CTCTCGGCATGGACGAGCTGTACAAGTAATGAAAGCTT

5.3 Gene encoding His6-SilicaL2-Hydrolase36P5-GFP in pAR2000

The gene for the His-tag is shown in blue, the gene for the Silica L2 tag (rplB) is shown in orange, the gene for the optimized hydrolase mutant 36P5 is shown in red, the flexible linker in purple, and GFP in green. Restriction sites are underlined.

ATGCACCATCACCATCACCATACCGGTGCAGTTGTTAAATGTAAACCGACATCTCCGGGTCGTCGCCA
CGTAGTTAAAGTGGTTAACCCTGAGCTGCACAAGGGCAAACCTTTTGCTCCGTTGCTGGAAAAAAACA
GCAAATCCGGTGGTCGTAACAACAATGGCCGTATCACCACTCGTCATATCGGTGGTGGCCACAAGCA
GGCTTACCGTATTGTTGACTTCAAACGCAACAAAGACGGTATCCCGGCAGTTGTTGAACGTCTTGAGT
ACGATCCGAACCGTTCCGCGAACATCGCGCTGGTTCTGTACAAAGACGGTGAACGCCGTTACATCCTG
GCCCCTAAAGGCCTGAAAGCTGGCGACCAGATTCAGTCTGGCGTTGATGCTGCAATCAAACCAGGTA
ACACCCTGCCGATGCGCAACATCCCGGTTGGTTCTACTGTTCATAACGTAGAAATGAAACCAGGTAAA
GGCGGTCAGCTGGCACGTTCCGCTGGTACTTACGTTCAGATCGTTGCTCGTGATGGTGCTTATGTCACC
CTGCGTCTGCGTTCTGGTGAAATGCGTAAAGTAGAAGCAGACTGCCGTGCAACTCTGGGCGAAGTTGG

CAATGCTGAGCATATGCTGCGCGTTCTGGGTAAAGCAGGTGCTGCACGCTGGCGTGGTGTTCGTCCGA
CCGTTCGCGGTACCGCGATGAACCCGGTAGACCACCCACATGGTGGTGGTGAAGGTCGTAACTTTGGT
AAGCACCCGGTAACTCCGTGGGGCGTTCAGACCAAAGGTAAGAAGACCCGCAGCAACAAGCGTACT
GATAAATTCATCGTACGTCGCCGTAGCAAAGCTAGCCCGTTAAACCCCCATGTCGAAGCCCTGTTGCA
AATGATGGCACAAATGCCCGCACCCGATTTCTCTGTAGCAAATCCGGCTGAAATCCGAGCGGTCTTTG
ACAATCCAATGCCGCTGGCCGCGCCGCCTCAGGTTGCCCGGGTAGAGGATATTGCGATTTCCCTCGAC
GGGCGGGATCTTGATGCGCGGCTTTACGTGCCCGAAGACGCGGATGAGCGACCTGCACTGATGGTCTA
TTATCATGGCGGCGGTTGGGTCATCGGAACTTTGGATACCCACGACGGCACCTGCCGTGCTCTAGCGC
AAAAAAGTGGCTGCGCTGTTTTGTCGATCGCTTATCGGCTGGCACCGGAGTATCGCTATCCGGCGCCC
GCCGAAGATTGCTATGACGCGCTGGTCTGGGCCAAGCAAAATGCCGCAACTCTGGGTGTGGATGGCG
ATCGCCTTGCCGTTGGCGGTGATAGCGCAGGCGGCAATCTTGCGGCTGCCGTTGCCATAATGGCCCGC
GACCGGAATGGTCCCGCGCTTCGCCATCAGTTGCTCATTTATCCCGCAACCGACAATGATTTCACTCTT
GCCTCCTATGCCGAAAATGGCGGGGGCGAATATTATCTCAGTACCGATGGCTTGCGCTGGTTCTGGGG
CCATTATCTTGGCGATACGGCAGCAGAAGATGCGCCGCTTGCCGCCGTTCTGAATGTGGCGGATCTGT
CTGGCCTCGCGCCGGCCACGGTTATCACGGCGGAATATGACCCCTTGCGTGACGAGGGCATTGCCTAT
GCCAAAAAGCTTGATGCCGCAGGCGTGCCGGTTGATGCAGCGACGGCGCCTGGCATGATCCACGGTTT
CTTCAGCATGTTCGAAGCTGTGCCCGATAGTTGGGAATGGATAGAACGAGGTGCATCCAATCTTAAAA
GAGACCTCGCTGGCGCGCCTTCAGGTAGCTCTGGCACTTCAAGCGGTACTAGTATGGTGAGCAAGGGC
GAGGAGCTGTTCACCGGGGTGGTGCCCATCCTGGTCGAGCTGGACGGCGACGTAAACGGCCACAAGT
TCAGCGTGTCCGGCGAGGGCGAGGGCGATGCCACCTACGGCAAGCTGACCCTGAAGTTCATCTGCAC
CACCGGCAAGCTGCCCGTGCCCTGGCCCACCCTCGTGACCACCCTGACCTACGGCGTGCAGTGCTTCA
GCCGCTACCCCGACCACATGAAGCAGCACGACTTCTTCAAGTCCGCCATGCCCGAAGGCTACGTCCA
GGAGCGCACCATCTTCTTCAAGGACGACGGCAACTACAAGACCCGCGCCGAGGTGAAGTTCGAGGGC
GACACCCTGGTGAACCGCATCGAGCTGAAGGGCATCGACTTCAAGGAGGACGGCAACATCCTGGGGC
ACAAGCTGGAGTACAACTACAACAGCCACAACGTCTATATCATGGCCGACAAGCAGAAGAACGGCA
TCAAGGTGAACTTCAAGATCCGCCACAACATCGAGGACGGCAGCGTGCAGCTCGCCGACCACTACCA
GCAGAACACCCCCATCGGCGACGGCCCCGTGCTGCTGCCCGACAACCACTACCTGAGCACCCAGTCC
GCCCTGAGCAAAGACCCCAACGAGAAGCGCGATCACATGGTCCTGCTGGAGTTCGTGACCGCCGCCG
GGATCACTCTCGGCATGGACGAGCTGTACAAGTAATGAAAGCTT
